# Sir2 regulates selective autophagy in stationary-phase yeast cells

**DOI:** 10.15698/mic2026.01.864

**Published:** 2026-01-19

**Authors:** Ji-In Ryu, Juhye Jung, Jeong-Yoon Kim

**Affiliations:** Department of Microbiology and Molecular Biology, College of Bioscience and Biotechnology, Chungnam National University, Daejeon, Republic of Korea

**Keywords:** *Saccharomyces cerevisiae*, Sir2, autophagy, stationary phase, complex medium

## Abstract

Autophagy contributes to cellular homeostasis by degrading and recycling intracellular components, especially under nutrient-limited conditions. While autophagy is well characterized under acute starvation in synthetic media in *Saccharomyces cerevisiae*, its regulation during the stationary phase of prolonged growth in nutrient-rich complex media, when cells experience gradual metabolic shifts and sustained stress, remains poorly understood. In this study, we identified Sir2, an NAD ${}^{+}$ -dependent histone deacetylase, as a key suppressor of autophagy during the stationary phase in YPD complex medium. Using GFP-Atg8 processing as a readout of autophagic flux, we demonstrated that *SIR2* deletion led to sustained autophagy activation. Notably, Sir2 selectively inhibited mitophagy, pexophagy, and the Cvt pathway, while non-selective autophagy remained largely unaffected. Transcriptomic analysis revealed that Sir2 facilitates a coordinated entry into quiescence, in part by regulating ribosome biogenesis and nutrient-responsive pathways during the stationary phase. Mechanistically, Sir2 stabilized Ume6, a repressor of *ATG8* transcription, thereby limiting autophagic activity. Deletion of *SIR2* drastically increased the phosphorylation and stabilization of the mitochondrial receptor Atg32 during the stationary phase, leading to enhanced mitophagy. Additionally, we found that ROS generated by mitophagy enhanced autophagy through a positive feedback loop. Collectively, our findings establish Sir2 as a previously unrecognized regulator of selective autophagy during the stationary phase in complex medium and highlight how cells dynamically control organelle degradation to maintain viability under extended metabolic stress.

## INTRODUCTION

Autophagy is an evolutionarily conserved and fundamental process essential for maintaining cellular homeostasis 1, 2. It facilitates the degradation and recycling of damaged organelles, misfolded proteins, and other macromolecules, particularly under nutrient-deprived conditions or cellular stress 3–5. It supports energy production, protein quality control, and the removal of dysfunctional cellular components, thereby enhancing cell survival and adaptability 6–10. Autophagy proceeds through the formation of a phagophore, its expansion into an autophagosome, and fusion with the vacuole for cargo degradation 11. Atg8, a ubiquitin-like protein conjugated to autophagosomal membranes, is essential for autophagosome expansion and serves as a widely used marker of autophagic flux 11. Autophagy can be either non-selective, engulfing bulk cytoplasm, or selective, targeting defined substrates such as mitochondria (mitophagy), peroxisomes (pexophagy), or vacuolar enzymes (the Cvt pathway) 9, 12. Selective autophagy requires cargo receptors and the scaffold protein Atg11 9, 12. In the budding yeast *Saccharomyces cerevisiae*, autophagy is crucial for promoting survival and longevity during periods of nutrient scarcity 13, 14.

Yeast cells undergo distinct metabolic transitions through exponential growth, the post-diauxic phase, and finally the stationary phase. These transitions involve substantial metabolic reprogramming, making autophagy an essential mechanism for survival 15, 16. A key transition, the diauxic shift, occurs when yeast cells switch from fermentative growth to respiratory metabolism due to glucose depletion 17, 18. This phase triggers autophagy as part of the cellular adaptation to nutrient limitation, facilitating the degradation of non-essential organelles and proteins and providing precursors for biosynthesis and energy production 19.

Traditional studies of yeast autophagy have predominantly relied on synthetic media and acute starvation models, where rapid nutrient depletion triggers strong autophagy induction 20–22. While these models have been instrumental in elucidating core molecular mechanisms and identifying key autophagy-related genes, they primarily reflect short-term stress responses and may not fully capture the dynamics of autophagy regulation under physiological conditions. Autophagy can also be triggered by more subtle shifts in metabolic and nutritional states; for example, a switch from rich medium to minimal, non-nitrogen-starvation medium activates autophagy to maintain cellular homeostasis in response to medium composition changes and metabolic adjustments 23. In contrast to these acute or clearly defined nutritional shifts, yeast cells grown in complex medium (YPD) and entering the stationary phase experience gradual, multifaceted environmental changes, including progressive nutrient depletion, accumulation of metabolic byproducts, and oxidative stress. Such metabolic complexity demands a carefully balanced regulation of autophagy, preventing excessive degradation of essential organelles that could lead to metabolic failure and compromise long-term cell viability.

Sir2 (Silent Information Regulator 2), a conserved NAD ${}^{+}$ -dependent histone deacetylase, has diverse roles in cellular metabolism, stress responses, and aging 24–27. This study demonstrates that Sir2 plays a critical role in suppressing autophagy during prolonged culture in YPD complex medium, ensuring a balance between cellular energy demands and the controlled recycling of intracellular components. These findings provide critical insights into the condition-specific mechanisms of autophagy regulation and have broader implications for understanding cellular lifespan, metabolic adaptation, and stress resistance.

## RESULTS

### Sir2 represses stationary phase autophagy in YPD complex medium

**Figure 1:**
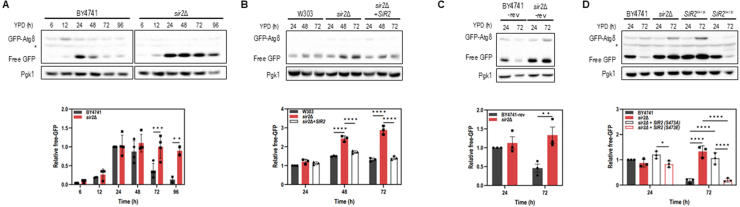
Sir2 represses autophagy during the stationary phase in YPD complex medium. **(A–C)** GFP–Atg8 processing in BY4741 **(A)**, W303 **(B)**, and BY4741-rev (a prototrophic revertant strain of BY4741) **(C)**. Wild-type and *sir2* Δ strains were cultured in YPD medium for up to 72 or 96 h. Cells were harvested at the indicated time points to assess autophagic activity. **(D)** Role of Sir2 phosphorylation in autophagy regulation. BY4741, *sir2* Δ , *SIR2* ${}^{S473A}$ (non-phosphorylable), and *SIR2* ${}^{S473E}$ (phospho-mimetic) strains expressing GFP-Atg8 were cultured in YPD medium for 24 and 72 hours. Cell lysates were analyzed by immunoblotting using anti-GFP and anti-Pgk1 antibodies. The asterisk indicates a nonspecific band. Quantification of free GFP release is shown below each blot. Data represent mean ± S.D. of at least three independent experiments. Statistical significance was assessed by two-way ANOVA with GraphPad Prism 10 (^⁎^*p* < 0.05, ^⁎⁎^*p* < 0.01, ^⁎⁎⁎^*p* < 0.001, ^⁎⁎⁎⁎^*p* < 0.0001).

To investigate whether Sir2 regulates autophagy during prolonged culture, we monitored GFP-Atg8 processing in WT and *sir2* Δ strains grown in complex (YPD) and synthetic complete (SC) media. When the N-terminus of Atg8 is tagged with GFP, the relatively stable GFP accumulates in the vacuole and can be quantified as a readout of autophagic activity. During the exponential phase, neither WT (BY4741) nor *sir2* Δ exhibited GFP-Atg8 processing. After the diauxic shift, both strains showed increased GFP-Atg8 processing; however, in YPD medium, WT cells showed a decline in processing during the stationary phase (48 h and 72 h), while *sir2* Δ maintained high levels (Figure 1**A**). This pattern was also observed in the W303 background (Figure 1**B**). To rule out the possibility that auxotrophic markers present in these laboratory backgrounds might themselves induce or indirectly influence autophagy phenotypes, we constructed a prototrophic revertant strain of BY4741 (Table S1). This strain reproduced the same pattern—low autophagy in WT and sustained autophagy in *sir2* Δ during the stationary phase in YPD—confirming that the observed phenotype is not due to auxotrophic background effects but reflects a genuine consequence of Sir2 loss (Figure 1**C**).

In contrast, in SC medium, GFP-Atg8 processing increased after the diauxic shift and decreased similarly in both strains during the stationary phase (Supplemental Figure S1A). Under acute nitrogen or carbon starvation, autophagy is known to be rapidly induced and to reach maximal levels within 4–6 h. Accordingly, we analyzed cells at 5 h, a representative peak time point at which differences would be expected if Sir2 were involved. At this stage, processing was comparable between WT and *sir2* Δ (Supplemental Figures S1B, S1C), indicating that Sir2 does not influence starvation-induced bulk autophagy in SC medium. These findings suggest that the regulatory role of Sir2 is specific to the stationary-phase program in YPD rather than to acute starvation responses. Given that Sir2 activity can be modulated by posttranslational modifications 28, 29, and its phosphorylated form is present during the stationary phase (Supplemental Figure S2), we tested the role of phosphorylation at S473 in autophagy regulation. The phospho-mimetic mutant SIR2-S473E restored the WT phenotype, whereas the non-phosphorylatable SIR2-S473A resembled *sir2* Δ in its elevated autophagic activity (Figure 1**D**). Together, these results suggest that Sir2 specifically limits autophagy during the stationary phase in YPD medium.

### Sir2 suppresses selective autophagy during the stationary phase

**Figure 2:**
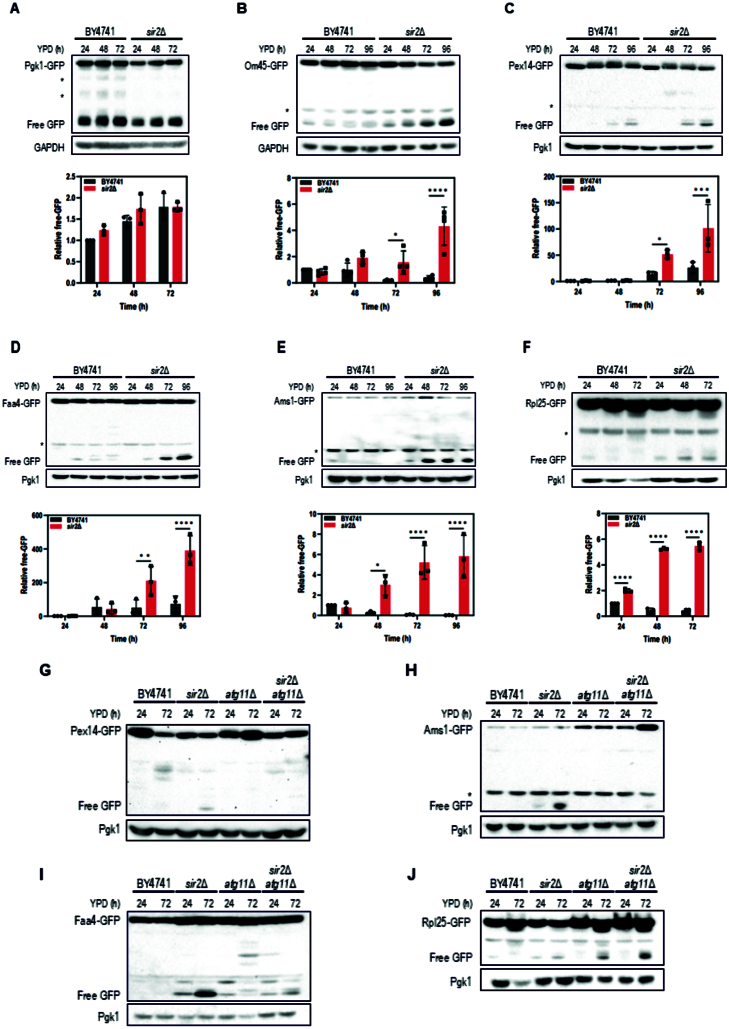
Sir2 represses selective autophagy during the stationary phase. **(A-F)** BY4741 wild-type and *sir2* Δ strains expressing GFP-tagged markers for various organelles or proteins - Pgk1 (cytosol; **A**), Om45 (mitochondria; **B**), Pex14 (peroxisomes; **C**), Faa4 (lipid droplets; **D**), Ams1 (cytosol to vacuole transport; **E**), or Rpl25 (ribosomes; **F**) - were cultured in YPD for up to 72 or 96 hours. Asterisks indicate non-specific bands. Quantification of free GFP release is shown below each blot. Data represent mean ± S.D. of at least three independent experiments. For statistical analysis, the *p*-values were calculated using two-way ANOVA (^⁎^*p* < 0.05, ^⁎⁎^*p* < 0.01, ^⁎⁎⁎^*p* < 0.001, ^⁎⁎⁎⁎^*p* < 0.0001). **(G–J)** BY4741 wild-type, *sir2* Δ *, atg11* Δ *, and sir2* Δ *atg11* Δ mutant strains expressing Pex14-GFP **(G)**, Ams1-GFP **(H)**, Faa4-GFP **(I)**, or Rpl25-GFP **(J)** were cultured in YPD medium for up to 72 hours. Cell lysates were prepared at the indicated time points for immunoblot analysis with anti-GFP or anti-Pgk1 antibodies.

Since Atg8 is incorporated into autophagosome membranes in both non-selective and selective autophagy, we investigated which type of autophagy is regulated by Sir2. We monitored GFP-processing in various GFP-tagged components (Om45, Pex14, Faa4, Ams1, and Rpl25) and GFP-tagged cytosolic proteins Pgk1. Pgk1-GFP processing showed no significant difference between WT (BY4741) and *sir2* Δ during the stationary phase, indicating that Sir2 does not regulate non-selective autophagy (Figure 2**A**). The same result was confirmed in the W303 strain background (Supplemental Figure S3A). Although Pgk1-GFP showed strong free-GFP accumulation at 48 h and 72 h, this likely reflects the cumulative stability of free GFP derived from the degradation of abundant Pgk1 during earlier peak activity rather than high autophagic activity at those late time points. To further confirm that Sir2 does not affect bulk autophagy, we also monitored processing of another cytosolic marker, Pgi1-GFP. Similar to Pgk1-GFP, Pgi1-GFP processing was comparable between WT and *sir2* Δ cells (Supplemental Figure S3B), providing independent support that bulk autophagy proceeds normally in the absence of Sir2. In contrast, Om45-GFP, Pex14-GFP, and Faa4-GFP showed increased GFP processing in *sir2* Δ , suggesting that Sir2 suppresses mitophagy, pexophagy, and lipophagy during the stationary phase (Figure 2**B**–2**D**). Additionally, Ams1-GFP, a cargo of the cytoplasm-to-vacuole targeting (Cvt) pathway, exhibited enhanced GFP processing in *sir2* Δ (Figure 2**E**). Ribophagy was also somewhat affected by Sir2 deficiency (Figure 2**F**). These findings suggest that Sir2 broadly suppresses multiple forms of selective autophagy during the stationary phase.

Most types of selective autophagy require the scaffold protein Atg11 to transport cargo to the pre-autophagosomal structure (PAS). Since our data showed that multiple forms of selective autophagy are induced in *sir2* Δ during the stationary phase, we investigated whether Sir2 regulates autophagy via Atg11. To address this, we first examined whether the Atg11 expression level is altered in *sir2* Δ cells. However, no significant changes were observed in the mRNA and protein levels of Atg11 (Supplemental Figures S4A, S4B). Furthermore, Atg11 localization was unchanged between WT and *sir2* Δ (Supplemental Figure S4C). We then assessed the processing of GFP-tagged cargo proteins representing distinct autophagy pathways with varying dependencies on Atg11 to explore the relationship between Sir2 and selective autophagy. Pex14–GFP was cleaved in *sir2* Δ cells but not in the *sir2* Δ *atg11* Δ double mutant (Figure 2**G**), demonstrating that Sir2 normally suppresses Atg11-dependent pexophagy under our conditions. In the Cvt pathway, which is strictly dependent on Atg11 30, *sir2* Δ cells exhibited clear Ams1–GFP processing, which was almost abolished in the *sir2* Δ *atg11* Δ double mutant (Figure 2**H**), indicating that loss of Sir2 permits Atg11-dependent degradation of Ams1. In the case of lipophagy, Faa4–GFP cleavage was reduced but not completely abolished in *sir2* Δ *atg11* Δ cells (Figure 2**I**), consistent with previous studies showing that lipophagy is only partially dependent on Atg11 31 and that a subset of events proceeds through Atg11-independent mechanisms 32. Thus, Sir2 appears to negatively regulate Atg11-dependent lipophagy under our conditions. In contrast, *SIR2* deletion increased Rpl25-GFP processing, though to a lesser extent than in *atg11* Δ . Notably, *sir2* Δ *atg11* Δ double mutants exhibited further enhanced Rpl25-GFP processing compared to *atg11* Δ alone (Figure 2**J**). Since Sir2 is not involved in bulk autophagy, and Rpl25-GFP degradation can occur through both selective (ribophagy) and non-selective pathways 33, these results suggest that *SIR2* deletion enhances ribosomal turnover through an Atg11-independent route, the nature of which remains to be defined. Together, these findings demonstrate that Sir2 inhibits multiple forms of selective autophagy during the stationary phase, and that its regulatory effect occurs independently of Atg11, supporting a role for Sir2 in autophagy regulation through alternative mechanisms.

### Deletion of *SIR2* disrupts coordinated entry into quiescence during the stationary phase

**Figure 3:**
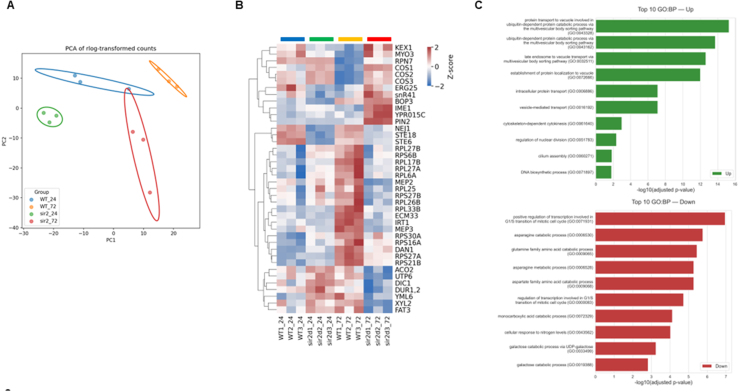
Sir2-dependent transcriptional reprogramming during the stationary phase. **(A)** Principal Component Analysis (PCA) of RNA-seq data from three independent biological replicates per condition. PC1 distinguishes 24 h from 72 h samples, reflecting temporal changes, while PC2 separates WT and *sir2* Δ strains, highlighting genotype-specific variation. **(B)** Heatmap of 40 genes with significant genotype × time interaction effects identified by likelihood ratio test (LRT) in DESeq2 (adjusted *p* < 0.05). Data are shown as row-normalized Z-scores of variance-stabilized counts. For each gene (row), expression values were centered on the mean and scaled by the standard deviation across all samples to highlight relative differences between conditions. Red indicates higher relative expression and blue indicates lower relative expression within each gene’s distribution. **(C)** Gene Ontology (GO) enrichment analysis of 265 genes differentially expressed in *sir2* Δ compared to WT at 72 h. Differential expression was determined using the Wald test in DESeq2 with adjusted *p* < 0.05 as the cutoff.

To investigate the molecular mechanisms underlying altered autophagy regulation in *sir2* Δ cells, we examine global transcriptional changes using RNA-seq. Principal component analysis (PCA) of rlog-transformed expression values revealed clear clustering of the four experimental groups (Figure 3**A**). PC1 accounted for temporal variation, distinctly separating 24 h and 72 h samples, while PC2 reflected genotypic differences between WT and *sir2* Δ . Notably, *sir2* Δ samples at 72 h formed a distinct cluster with minimal overlap with other groups, indicating marked transcriptomic divergence specifically during the stationary phase. To identify genes contributing to genotype-specific transcriptional changes over time, we applied a likelihood ratio test (LRT) to detect significant genotype × time interaction effects. This analysis identified 40 differentially expressed genes with distinct expression trajectories between WT and *sir2* Δ (adjusted *p*<0.05). Heatmap visualization of row-normalized z-scores revealed clear clustering of genes with similar relative expression patterns, with differences between WT and *sir2* Δ becoming most pronounced at 72 h (Figure 3**B**). At this time point, WT cells maintained relatively higher expression of ribosomal protein genes and factors involved in translation and mitochondrial function compared to *sir2* Δ cells, suggesting an enhanced repression of basal protein synthesis and metabolic support pathways in *sir2* Δ cells. In contrast, *sir2* Δ cells displayed elevated expression of vacuolar and membrane trafficking genes, developmental regulators of mating and meiosis, and stress-related factors, indicating untimely activation of pathways that are normally restrained in WT.

To complement this temporal analysis, we next performed a direct comparison between WT and *sir2* Δ specifically at 72 h (Supplemental Figure S6, Table S2). This endpoint analysis revealed robust upregulation of vacuolar sorting and multivesicular body (MVB) pathway genes in *sir2* Δ relative to WT, particularly members of the COS family (*COS1–8*, *COS10*, and *COS12*), which are known Sir2 targets 34 (Figure 3**C**, Supplemental Figure S5). Additional upregulated genes included those associated with nuclear division, cytokinesis, and DNA biosynthesis, suggesting inappropriate continuation of cell cycle-related programs. Conversely, genes involved in amino acid catabolism, nitrogen response, and galactose metabolism were downregulated in *sir2* Δ , indicating impaired nutrient-responsive transcription and quiescence entry. Together, these findings demonstrate that Sir2 fine-tunes transcriptional reprogramming during the stationary phase by preserving essential ribosomal and mitochondrial functions while suppressing mis-timed induction of vacuolar, developmental, and stress-response programs.

### Sir2 regulates Ume6 at the protein level to control *ATG8* transcription

**Figure 4:**
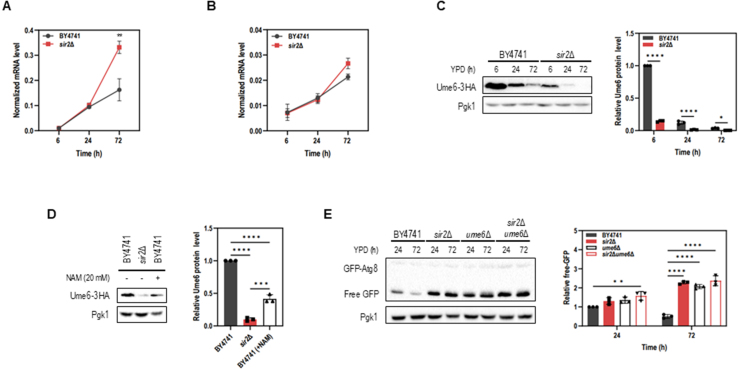
Sir2 regulates Ume6 at the protein level to control *ATG8* transcription. **(A, B)** Quantitative RT-PCR analysis of *ATG8***(A)***,* and *UME6***(B)** mRNA levels in WT and *sir2* Δ strains cultured in YPD medium for 6, 24, and 72 hours. Expression levels were normalized to *ACT1* mRNA levels. **(C)** Protein levels of Ume6-3HA in BY4741 and *sir2* Δ strains cultured in YPD medium for up to 72 hours. **(D)** Ume6-3HA protein levels in BY4741 and *sir2* Δ strains cultured for 6 hours in the presence or absence of 20 mM nicotinamide (NAM), a Sir2 inhibitor. **(E)** Autophagic activity in BY4741, *sir2* Δ , *ume6* Δ , and *sir2* Δ *ume6* Δ strains expressing GFP-Atg8 cultured in YPD medium for up to 72 hours. Cells were collected at the indicated time points. All samples were subjected to immunoblot analysis using anti-GFP, anti-HA, or anti-Pgk1 antibodies. Quantification of Ume6-3HA and free GFP release is shown next to each blot. Data represent the mean ± S.D. from at least three independent experiments. Statistical significance was determined using one-way or two-way ANOVA (^⁎^*p* < 0.05, ^⁎⁎^*p* < 0.01, ^⁎⁎⁎^*p* < 0.001, ^⁎⁎⁎⁎^*p* < 0.0001).

Although our transcriptomic analysis revealed broad dysregulation of metabolic and vacuolar sorting pathways in *sir2* Δ cells, these changes alone did not fully explain the enhanced selective autophagy phenotypes observed by Atg11-dependent autophagy. Because Atg8 is both essential for autophagosome formation and widely used as an autophagy reporter, we asked whether *ATG8* expression itself might be altered in *sir2* Δ . Indeed, *ATG8* mRNA levels were significantly elevated during the stationary phase at 72 h (Figure 4**A**), which may contribute to the sustained autophagy observed in *sir2* Δ cells.

We next hypothesized that Sir2 regulates *ATG8* transcription through the Ume6–Sin3–Rpd3 complex, a known repressor of *ATG8* expression 35. To test this, we examined Ume6 expression at both the mRNA and protein levels. *UME6* transcript levels remained similar between WT and *sir2* Δ cells at 6 h, 24 h, and 72 h (Figure 4**B**), whereas Ume6 protein was markedly reduced in *sir2* Δ (Figure 4**C**). Similarly, treatment with nicotinamide, a Sir2 inhibitor, also decreased Ume6 protein level (Figure 4**D**), suggesting that Sir2 plays a role in maintaining Ume6 stability. To assess the functional consequences of Ume6 depletion, we examined autophagy induction in Ume6-depleted cells. We found that *UME6* deletion enhanced GFP-Atg8 processing in the stationary phase (Figure 4**E**), indicating increased autophagic activity. However, we also noted that Ume6 protein level naturally declined in WT cells during the stationary phase, with markedly reduced levels at 72 h (Figure 4**C**). Despite this reduction, autophagic activity remained low at this time point in WT cells (Figure 4**E**). This observation suggests that decreased Ume6 abundance alone is insufficient to trigger significant autophagy induction under these conditions. Together, these findings indicate that while Sir2 supports Ume6 protein stability to maintain repression of *ATG8* transcription, the enhanced autophagy observed in *sir2* Δ cells during the stationary phase cannot be solely attributed to Ume6 depletion and likely involves additional Sir2-dependent regulatory mechanisms.

### Sir2 deficiency results in increased ROS levels during the stationary phase

**Figure 5:**
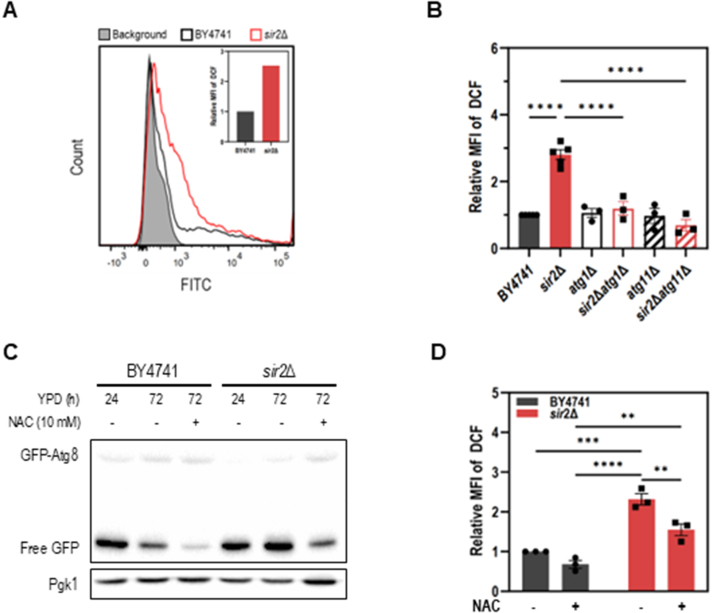
Sir2 deficiency increases ROS levels during the stationary phase. **(A)** ROS levels in BY4741 and *sir2* Δ strains at 72 h of culture, measured by 2 ${}^{\prime }$ ,7 ${}^{\prime }$ -dichlorodihydrofluorescein diacetate (DCF) fluorescence using FACS (excitation: 488nm; emission: 515–545nm). **(B)** ROS levels in BY4741, *sir2* Δ , *atg1* Δ , *sir2* Δ *atg1* Δ , *atg11* Δ , and *sir2* Δ *atg11* Δ strains at 72 h of culture, measured using a microplate reader (excitation: 485nm; emission: 535nm). Data represent the mean ± S.D. from at least three independent experiments. For statistical analysis, the *p*-values were calculated using one-way ANOVA (^⁎⁎⁎⁎^*p*<0.0001). MFI, mean fluorescence intensity. **(C)** Effect of N-acetylcysteine (NAC) on autophagic activity in BY4741 and *sir2* Δ strains expressing GFP-Atg8. Cells were cultured in YPD for 24 h, treated with 10 mM NAC, and incubated up to 72 h. Cell lysates were analyzed by immunoblotting with anti-GFP or anti-Pgk1 antibodies. **(D)** ROS levels in BY4741 and *sir2* Δ strains cultured with or without 10 mM NAC up to 72 h, measured using a microplate reader (excitation: 485 nm; emission: 535 nm). Data represent the mean ± S.D. from at least three independent experiments. Statistical significance was determined using two-way ANOVA (^⁎⁎^*p* < 0.01, ^⁎⁎⁎^*p* < 0.001, ^⁎⁎⁎⁎^*p* < 0.0001).

Since Ume6-mediated *ATG8* transcription alone may not fully explain the elevated autophagy in *sir2* Δ cells, we explored whether other Sir2-related mechanisms contribute to the enhanced autophagy, focusing on oxidative stress, a condition closely linked to autophagy regulation. FACS analysis revealed a two-fold increase in reactive oxygen species (ROS) in *sir2* Δ cells compared to WT cells, suggesting that *SIR2* deletion leads to elevated ROS levels (Figure 5**A**). To determine whether this ROS accumulation is associated with enhanced autophagy, we measured ROS levels in autophagy-deficient mutants. Deletion of *ATG1,* which blocks autophagy initiation, significantly reduced ROS levels in *sir2* Δ cells, suggesting that autophagy contributes to ROS accumulation. Similarly, deletion of *ATG11*, which disrupts selective autophagy, led to a comparable reduction in ROS (Figure 5**B**), indicating that selective autophagy plays a substantial role in ROS generation in *sir2* Δ cells. Conversely, as ROS can also act as a signaling molecule to promote autophagy 36, we tested whether reducing ROS levels would suppress autophagy in *sir2* Δ cells during the stationary phase. Indeed, treatment with N-acetyl cysteine (NAC), a well-established ROS scavenger, led to a marked decrease in autophagic activity and ROS level in *sir2* Δ cells (Figure 5**C**, 5**D**). Collectively, these findings suggest that autophagy, particularly selective autophagy, serves as a major source of ROS in the absence of Sir2, and that ROS generated through this process further promotes autophagy, establishing a potential positive feedback loop during the stationary phase of *sir2* Δ cells.

### Sir2 deficiency leads to Atg32 phosphorylation and stability during the stationary phase

**Figure 6:**
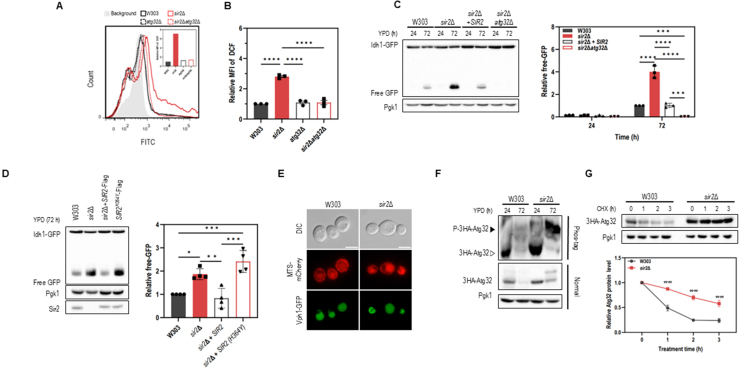
Sir2 regulates Atg32 phosphorylation and stability during the stationary phase. **(A)** ROS levels in W303, *sir2* Δ , *atg32* Δ , and *sir2* Δ *atg32* Δ strains at 72 h of culture measured by 2 ${}^{\prime }$ ,7 ${}^{\prime }$ -dichlorodihydrofluorescein diacetate (DCF) fluorescence using FACS (excitation: 488nm; emission: 515–545nm). **(B)** ROS levels in W303, *sir2* Δ , *atg32* Δ , and *sir2* Δ *atg32* Δ strains at 72 h of culture. DCF fluorescence intensity was measured using a microplate reader (excitation: 485 nm; emission: 535 nm). **(C)** Idh1-GFP processing in W303, *sir2* Δ , *sir2* Δ +*SIR2*, and *sir2* Δ *atg32* Δ strains cultured in YPD medium for up to 72 hours. **(D)** Idh1-GFP processing in W303, *sir2* Δ , *SIR2*-Flag rescue, and *SIR2* ${}^{\mathrm{H364Y}}$ -Flag rescue strains cultured in YPD medium for 72 hours. Cell lysates were analyzed by immunoblotting using anti-GFP, anti-Sir2, and anti-Pgk1 antibodies. **(E)** Mitochondrial protein accumulation in the vacuole. W303 and *sir2* Δ strains expressing MTS-mCherry and Vph1-GFP, which was used as a vacuolar marker, were cultured in YPD medium for 72 hours. Localization of mCherry and GFP signals was visualized by fluorescence microscopy. DIC, differential interference contrast. Scale bar: 5 μ m. **(F)** Atg32 phosphorylation in W303 and *sir2* Δ strains analyzed by Phos-tag SDS-PAGE and immunoblotting. Arrowheads indicate phosphorylated (black) and non-phosphorylated (white) forms of 3HA-Atg32. **(G)** Atg32 stability in W303 and *sir2* Δ strains. Cells were cultured in YPD medium for 24 hours and treated with cycloheximide (CHX, 1 mM) for 1, 2, or 3 hours. Cell lysates were analyzed by immunoblotting using anti-HA and anti-Pgk1 antibodies. Quantification of free GFP release and 3HA-Atg32 is shown below or next to each blot. Data represent the mean ± S.D. from at least three independent experiments. Statistical significance was determined using one-way or two-way ANOVA, analyzed with GraphPad Prism 10. (^⁎^*p* < 0.05, ^⁎⁎^*p* < 0.01, ^⁎⁎⁎^*p* < 0.001, ^⁎⁎⁎⁎^*p* < 0.0001).

To determine whether ROS accumulation in *sir2* Δ cells is specifically linked to mitophagy, which is closely associated with ROS production, we performed a series of targeted experiments. All subsequent analyses were conducted using the W303 strain, a widely used background in mitochondrial research 37, 38. Strikingly, ROS levels were markedly reduced in *sir2* Δ *atg32* Δ double mutants, in which mitophagy is blocked due to deletion of *ATG32* (Figure 6**A**, 6**B**). This finding suggests that the elevated mitophagy is a primary contributor to ROS accumulation in *sir2* Δ cells. Moreover, the initial ROS increase may promote additional selective autophagy, establishing a positive feedback loop that amplifies both mitophagy and oxidative stress. Given these findings, we further examined mitophagy dynamics and mitochondrial homeostasis in detail. To directly monitor mitophagy, we tagged GFP to both an outer membrane protein (Om45) and a matrix protein inside mitochondria (Idh1). In *sir2* Δ cells, both Om45-GFP and Idh1-GFP showed enhanced GFP processing during the stationary phase, indicating increased mitophagy activity (Figure 6**C**, Supplemental Figure S7A). This increase was suppressed by reintroducing wild-type *SIR2*, but not by expressing a catalytically inactive Sir2 mutant (SIR2-H364Y), underscoring the importance of Sir2’s enzymatic activity in regulating mitophagy (Figure 6**D**). Additional evidence of increased mitophagy in *sir2* Δ cells came from the enhanced vacuolar accumulation of MTS-tagged mCherry, a reporter of mitochondrial delivery to the vacuole (Figure 6**E**), together with consistently lower levels of the outer membrane protein Por1 over time (Supplemental Figure S7B).

Because Atg32 phosphorylation is required for its interaction with Atg11 and subsequent mitochondrial degradation, we assessed Atg32 phosphorylation status in *sir2* Δ cells. Phosphorylated Atg32 levels were significantly elevated in *sir2* Δ compared to wild-type (Figure 6**F**), despite a reduction in *ATG32* mRNA levels (Supplemental Figure S7C). Moreover, total Atg32 protein was markedly more stable in *sir2* Δ cells (Figure 6**G**). These results suggest that Sir2 normally limits Atg32 activation and promotes its turnover, and that its absence leads to persistent Atg32 activity, resulting in excessive mitophagy. Collectively, these findings demonstrate that deletion of *SIR2* enhances Atg32 phosphorylation and stability, resulting in sustained mitophagy and elevated ROS generation. This promotes a self-amplifying cycle of selective autophagy and oxidative stress, particularly during the stationary phase.

## DISCUSSION

Autophagy is essential for maintaining cellular homeostasis and survival under starvation or stress conditions. However, its timing and extent must be precisely regulated, as excessive or prolonged autophagy can lead to the degradation of vital cellular components and compromise cell viability. In this study, we demonstrate that Sir2 functions as a key regulator of selective autophagy during the stationary phase in *Saccharomyces cerevisiae* grown in complex medium, where prolonged metabolic stress imposes distinct physiological challenges compared to acute starvation models.

Autophagy has traditionally been studied using synthetic media and acute nutrient deprivation, which trigger rapid and transient activation of the autophagic machinery 39–41. These conditions have provided valuable insight into the core mechanisms of autophagy. However, they may not fully reflect the cellular responses during prolonged growth in rich media, such as YPD, where cells undergo gradual nutrient depletion, metabolic reprogramming, and oxidative stress accumulation. Under these conditions, autophagy is required not just for acute adaptation but for sustained recycling and metabolic support, enabling cells to survive in a quiescent state. This prolonged survival capacity is reflected in chronological lifespan, which is significantly shortened in autophagy-deficient mutants (e.g., *atg1* Δ ), emphasizing the protective role of autophagy under extended nutrient limitation 42.

Although the TOR and cAMP-PKA pathways have been extensively studied as key regulators of autophagy 43–46, the role of Sir2 in autophagy regulation has remained largely unexplored. Previous studies suggested a potential link between Sir2 and autophagy under specific stress contexts, such as α -synuclein toxicity 47. Moreover, homologs of Sir2 in higher organisms, such as *Drosophila* Sir2 and mammalian SIRT1, have been shown to promote autophagy by regulating the transcription of autophagy-related genes or deacetylating key autophagic proteins 48, 49. These findings support a generalized view of sirtuins as positive regulators of autophagy. However, our data challenge this notion and reveal that yeast Sir2 acts as a context-dependent suppressor of selective autophagy. We found that Sir2 selectively inhibits mitophagy, pexophagy, and lipophagy during the stationary phase, while non-selective autophagy remains largely unaffected. This selective restraint likely serves to prevent excessive degradation of critical organelles, preserving essential functions needed for long-term survival under metabolic stress.

Mechanistically, Sir2 regulates autophagy via multiple distinct pathways. First, it stabilizes the transcriptional repressor Ume6, which in turn suppresses *ATG8* expression and limits autophagosome formation (Figure 4). Second, Sir2 suppresses mitophagy by indirectly regulating the phosphorylation and stabilization of the mitochondrial receptor Atg32 (Figure 6). Atg32 is activated by CK2-dependent phosphorylation, which enhances its interaction with Atg11 and promotes recruitment of the core autophagic machinery 50, 51. Our findings suggest that Sir2 may interfere with this activation cascade, thereby preventing excessive mitophagy. In this context, an intriguing possibility is that Sir2 may communicate with the mitophagy machinery through crosstalk with CK2. CK2 is known to phosphorylate both Atg32 50 and Sir2 29, suggesting a potential bidirectional regulatory relationship in which Sir2 may also influence CK2 activity. Such crosstalk could provide a route by which Sir2 indirectly modulates Atg32-dependent mitophagy. However, the intermediate signaling steps linking nuclear Sir2 to CK2, and CK2 to Atg32, remain unresolved, and future studies will be required to clarify these mechanistic connections. Similarly, the mechanism by which Sir2 stabilizes Ume6 is also unclear. Ume6 is a target of the ubiquitin–proteasome mediated degradation 52. It is therefore possible that Sir2 influences Ume6 stability either by directly deacetylating Ume6 or by regulating E3 ubiquitin ligases or other components of the protein degradation machinery.

A particularly striking consequence of Sir2 deficiency is an apparent ROS–autophagy feedback loop. Under normal conditions, autophagy mitigates oxidative stress by removing damaged organelles 51, 53. However, in *sir2* Δ stationary-phase cells, dysregulated mitophagy instead increases ROS levels, creating a self-amplifying cycle that accelerates organelle degradation and may ultimately compromise cellular viability (Figures 5, 6). These findings align with observations from mammalian studies, where pharmacological inhibition of mitochondrial respiratory chain complex I enhanced PINK1-dependent mitophagy while simultaneously increasing cellular ROS. Conversely, genetic inhibition of autophagy or mitophagy reduced ROS levels, suggesting that mitophagy can function as a source of oxidative stress under certain conditions 54. Collectively, these results highlight a critical role for Sir2 in preventing ROS–mitophagy feedback and maintaining mitochondrial integrity and redox balance during nutrient limitation.

In conclusion, this study identifies Sir2 as a central regulator that prevents excessive organelle degradation in stationary-phase yeast cells grown in a complex medium. Sir2 limits autophagic activity by stabilizing Ume6 to repress ATG8 transcription and by suppressing Atg32 activation, thereby preventing excessive mitophagy. By moderating the autophagy–ROS feedback loop, Sir2 helps maintain organellar integrity and ensures cellular viability during extended nutrient stress. These findings not only broaden our understanding of sirtuin-mediated stress responses in yeast but also suggest that conserved mechanisms may exist in higher eukaryotes, where sirtuins regulate autophagy, redox homeostasis, and aging.

## MATERIALS AND METHODS

### Yeast strains and growth conditions

The yeast strains used in this study are listed in Table S1. Yeast cells were cultured in either rich (YPD) medium, consisting of 20 g/L Bacto peptone (Becton Dickinson), 10 g/L yeast extract (Becton Dickinson), and 20 g/L dextrose (Junsei), or synthetic complete (SC) medium, containing 0.67 g/L yeast nitrogen base without amino acids (Becton Dickinson), an amino acid mixture, and 20 g/L dextrose (Junsei). Autophagy was induced by nitrogen or carbon starvation using SD-N medium (0.17 g/L yeast nitrogen base without amino acids and ammonium sulfate (Becton Dickinson) with 20 g/L dextrose (Junsei)) and SC-C medium (0.67 g/L yeast nitrogen base without amino acids (Becton Dickinson) and an amino acid mixture without a carbon source), respectively. Synthetic drop-out (SD/-) medium, supplemented with 0.67 g/L yeast nitrogen base without amino acids (Becton Dickinson) and an amino acid mixture lacking uracil or histidine, was used for selecting yeast transformants. When necessary, transformants were plated onto solid medium containing 5-fluoroorotic acid (5-FOA, 1 mg/mL) to select for the loss of the *URA3* marker.

### Western blotting

Yeast cells were grown in each medium at 30 ${}^{\circ }$ C, harvested at exponential phase (6 h), post diauxic phase (24 h), and stationary phase (72 h) before being stored at − 80 ${}^{\circ }$ C. Protein extracts were prepared using the trichloroacetic acid (TCA) precipitation method. Cells were resuspended in an ice-cold NaOH solution (0.2 M NaOH and 0.2% β -mercaptoethanol) and incubated on ice for 10 min, followed by the addition of 10% TCA and further incubation on ice for 10 min. Samples were centrifuged at 13,000 rpm, 4 ${}^{\circ }$ C, for 15 min, and the supernatant was discarded. The resulting pellet was resuspended in sample loading buffer and 1 M Tris-HCl. Protein samples were separated on 8%–12% SDS-polyacrylamide gels and transferred to polyvinylidene fluoride (PVDF) membranes (Millipore). Membranes were probed with specific primary antibodies, followed by detection using HRP-conjugated secondary antibodies. The following primary antibodies were used: anti-GFP (1:2000; Santa Cruz Biotechnology), anti-Flag (1:2000; Sigma), anti-3HA (1:2,000; Santa Cruz Biotechnology), anti-Sir2 (1:200; Santa Cruz Biotechnology), anti-Por1 (1:2,000; Invitrogen), anti-Pgk1 (1:10,000; Abcam), and anti-GAPDH (1:10,000; Acris). Immunoreactivity was detected using enhanced chemiluminescence reagent (Thermo Scientific). Band intensity was quantified using Image Lab software (Bio-Rad).

### Phos-tag SDS-PAGE

To detect phosphorylated Sir2 and Atg32 proteins, total cell extracts were separated on 6% SDS-PAGE gels containing 25 mM Phos-tag (Wako) and 100 mM MnCl ${}_{2}$ , following the manufacturer’s instructions. The proteins were then analyzed by Western blotting using anti-Sir2 (1:200, Santa Cruz Biotechnology) and anti-3HA (1:2000, Santa Cruz Biotechnology) antibodies. Phosphorylated Sir2 and Atg32 were detected as upshifted bands in Phos-tag SDS-PAGE, and phosphorylation levels were determined by comparing the upshifted bands to their corresponding non-phosphorylated forms.

### Protein stability assay

To assess Atg32 stability, yeast cells expressing 3HA-Atg32 were cultured in YPD at 30 ${}^{\circ }$ C for 24 hours, then resuspended in fresh YPD containing 1 mM cycloheximide and incubated at 30 ${}^{\circ }$ C for 1, 2, or 3 hours. At each time point, a stop solution (200 mM sodium azide and 50mg/mL bovine serum albumin) was added on ice for 5minutes. Protein extracts were then prepared and analyzed by Western blotting using anti-HA antibodies to detect 3HA-Atg32.

### Measurement of intracellular ROS levels

Intracellular ROS levels were measured using 2 ${}^{\prime }$ ,7 ${}^{\prime }$ -dichlorodihydrofluorescein diacetate (DCF, Sigma). Yeast cells were cultured in YPD medium until the stationary phase (72 h), harvested by centrifugation at 3,000 rpm for 5 min, and washed twice with phosphate-buffered saline (PBS). Cells were then resuspended in PBS containing 0.2 mM DCF and incubated at 30 ${}^{\circ }$ C for 1 h, followed by two additional washes with PBS. For fluorescence quantification, cells were resuspended in PBS buffer, loaded into a black-walled microplate (SPL), and analyzed using a Varioskan Lux microplate reader with excitation at 485 nm and emission at 535 nm. Fluorescence intensity was normalized to OD600, and relative mean fluorescence intensity (MFI) was determined using the equation: Relative MFI = (F ${}_{\mathrm{test}}-{\text{F}}_{\mathrm{blank}}$ )/(F ${}_{\mathrm{control}}-{\text{F}}_{\mathrm{blank}}$ ), where F ${}_{\mathrm{test}}$ , F ${}_{\mathrm{blank}}$ , F ${}_{\mathrm{control}}$ represent the fluorescence readings from the *S. cerevisiae* mutant strains, the unstained *S. cerevisiae* and the wild-type *S. cerevisiae*. Additionally, fluorescence intensity was measured using FACS Canto II flow cytometer (Becton Dickinson) to assess ROS levels at the single-cell level.

### RNA-seq and data analysis

RNA-seq experiments were performed to examine transcriptional changes associated with *SIR2* deletion at 24 h and 72 h in wild-type and *sir2* Δ strains. Three independent biological replicates were prepared per condition. Total RNA was extracted using TRIzol reagent (Invitrogen) following the manufacturer’s protocol. RNA integrity was assessed with an Agilent 2100 Bioanalyzer, and RNA concentration was measured using an ND-2000 spectrophotometer (Thermo Scientific, DE, USA). Libraries were constructed using the QuantSeq 3 ${}^{\prime }$ mRNA-Seq Library Prep Kit (Lexogen, Austria) and sequenced on an Illumina NextSeq 500 platform to generate single-end 75 bp reads. Raw sequencing reads were quality-checked with FastQC (v0.11.9), trimmed for adapters using Cutadapt (v4.4), aligned to the *Saccharomyces cerevisiae* S288C reference genome (R64-3-1) with Bowtie2 55, and summarized at the gene level using Bedtools 56.

For downstream analysis, count data were processed with the DESeq2 package (v1.40.2) in R, interfaced via rpy2 (v3.5.17). Variance-stabilized counts were generated using DESeq2’s regularized log (rlog) transformation. Principal component analysis (PCA) was performed on the transposed rlog matrix (samples × genes) with scikit-learn (v1.4.2), and 95% confidence ellipses (approximated as two standard deviations) were calculated from each group’s sample covariance matrix. To identify genes with significant genotype × time interaction effects, a likelihood ratio test (LRT) was conducted by comparing a full model ( ∼ genotype + time + genotype:time) to a reduced model ( ∼ genotype + time). To assess transcriptional differences specifically at 72 h, pairwise differential expression analysis between WT and *sir2* Δ was performed using the Wald test. Genes with an adjusted *p*-value < 0.05 were considered significantly differentially expressed.

### Real-time PCR analysis

Total RNA was isolated using the NucleoSpin RNA kit (Macherey Nagel) and quantified by measuring absorbance at 260 nm. cDNA synthesis was performed from 1 μ g of total RNA using the ReverTra Ace qPCR RT kit (Toyoobo) according to the manufacturer’s instructions. Quantitative real-time PCR (qRT-PCR) was conducted using SYBR Green PCR mix and the CFX Connect Real-Time PCR System (Bio-Rad). Gene expression levels were normalized to *ACT1*, and relative expression was calculated using the comparative Ct ( Δ Δ Ct) method.

### Microscopy

To examine the localization of Atg11-GFP, Vph1-mCherry, Vph1-GFP, and MTS-mCherry, yeast cells expressing GFP and/or mCherry were cultured in YPD medium and harvested at the exponential phase (6 h), post-diauxic phase (24 h), and stationary phase (72 h). Cells were centrifuged at 3,000 rpm for 5 min, washed twice with PBS buffer, and resuspended in PBS. Fluorescent images were captured using an Olympus BX51 microscope with the following excitation/emission settings: GFP (460–490 nm/520 nm) and mCherry (530–550 nm/575 nm). The percentage of cells with vacuolar-targeted mitochondria and mitochondrial morphology was quantified using ImageJ (National Institutes of Health).

## AUTHORS CONTRIBUTIONS

JIR and JJ conducted experiments and wrote the manuscript; JYK conceived the concept, supervised the study, and wrote and edited the manuscript.

## SUPPLEMENTAL MATERIAL

All supplemental data for this article are available online at http://microbialcell.com/researcharticles/2026a-ryu-microbial-cell/. 

## CONFLICT OF INTEREST

The authors declare no conflict of interest.

## ABBREVIATIONS

cvt – cytoplasm-to-vacuole targeting

LRT – likelihood ratio test

NAC – N-acetyl-cysteine

PCA – principal component analysis

ROS – reactive oxygen species
