# Supplementary material for: Sir2 regulates selective autophagy in stationary-phase yeast cells
Source: Microb Cell. 2026 Jan 19;13:1–12. doi: 10.15698/mic2026.01.864 (PMC12826798; doi:10.15698/mic2026.01.864)
Supplement: Supplementary file 1 [file mic-13-001-s01.pdf]

Sir2 regulates selective autophagy in stationary-phase yeast cells

Ji-In Ryu[1,#], Juhye Jung[1,#], and Jeong-Yoon Kim[1,*]

[1]Department of Microbiology and Molecular Biology, College of Bioscience and Biotechnology, Chungnam National University, Daejeon, Republic of Korea

[#] Ji-In Ryu and Juhye Jung are equal contributors to this paper.

[*] Corresponding author: Jeong-Yoon Kim,

Department of Microbiology and Molecular Biology

College of Bioscience and Biotechnology

Chungnam National University

Daejeon 305-764, Republic of Korea

Tel: 82-10-9267-1388

E-mail: jykim@cnu.ac.kr

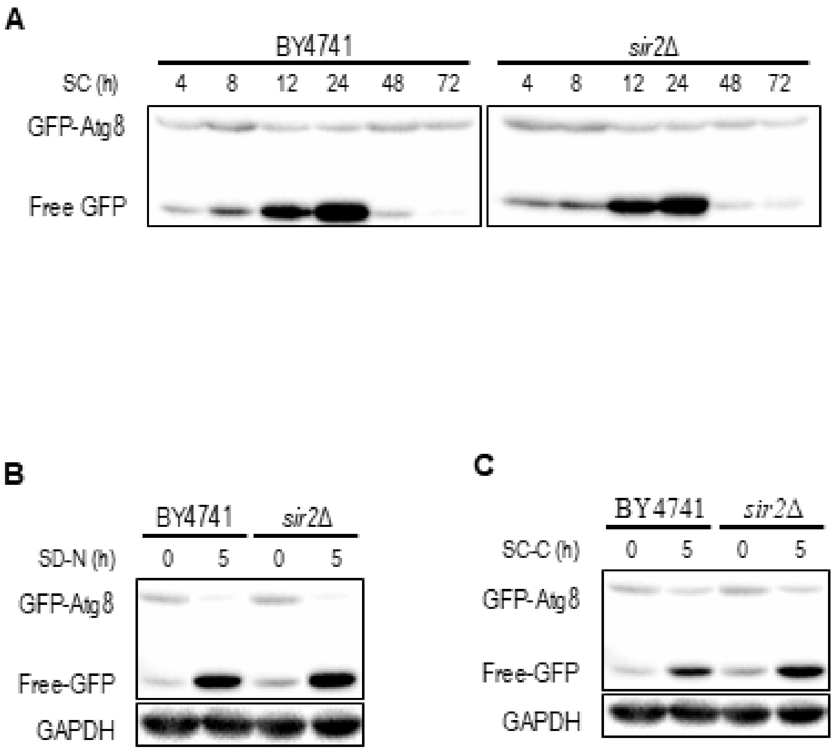

Supplementary Figure 1. (A) Autophagic activity in BY4741 wild-type and *sir2*Δ strains expressing GFP-Atg8 cultured in synthetic complete medium (SC) for up to 72 hours. Cells were collected at the indicated time points for analysis. (B, C) BY4741 and *sir2*Δ strains expressing GFP-Atg8 were cultured in YPD medium to mid-log phase, then shifted to SD-N (nitrogen starvation; B) or SC-C (carbon starvation; C) medium for 0 and 5 hours to induce acute autophagy.

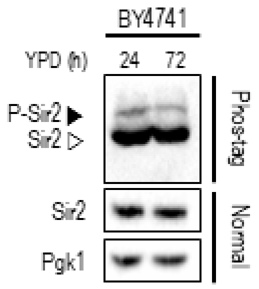

Supplementary Figure 2. Sir2 phosphorylation in BY4741 strains during the stationary phase. Cell lysates were analyzed by Phos-tag SDS-PAGE and immunoblotting using anti-Sir2 and anti-Pgk1 antibodies. Arrowheads indicate phosphorylated (black) and non-phosphorylated (white) forms of Sir2.

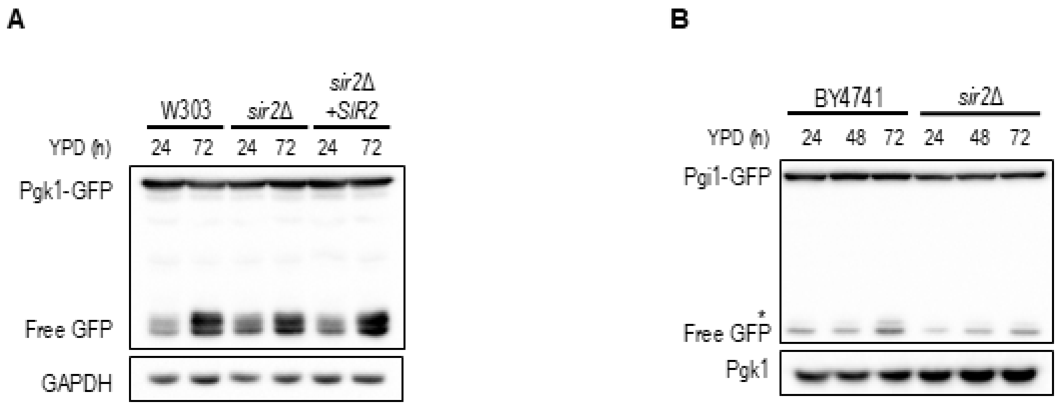

Supplementary Figure 3. (A) Bulk autophagy monitored by Pgk1-GFP processing in W303 wild-type, *sir2*Δ, and *SIR2*-rescue strains. (B) Bulk autophagy monitored by Pgi1-GFP in BY4741 and *sir2*Δ. Cell lysates collected at the indicated time points were analyzed by immunoblotting using anti-GFP to detect Pgk1-GFP or Pgi1-GFP. Anti-GAPDH was used as a loading control.

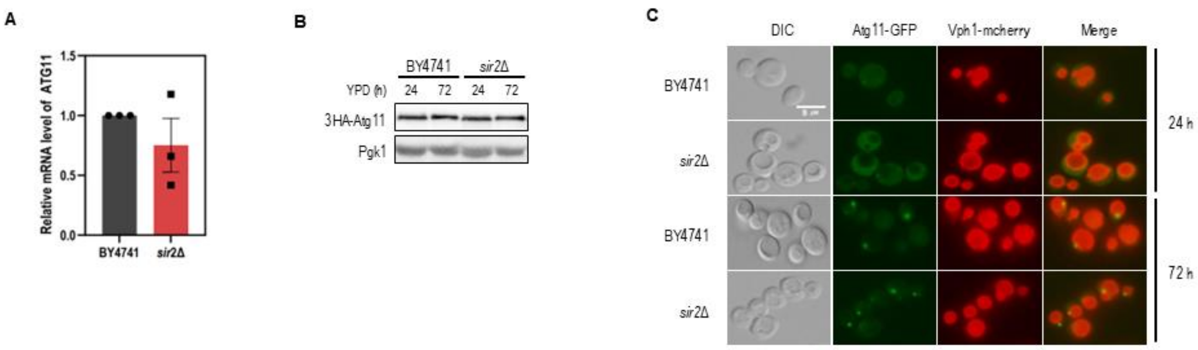

Supplementary Figure 4. Sir2 does not affect *ATG11* mRNA or protein levels, nor Atg11 localization. (A) Quantitative RT-PCR analysis of *ATG11* mRNA levels in WT and *sir2*Δ strains cultured in YPD medium for 72 hours. (B) Immunoblot analysis of 3HA-Atg11 protein levels in WT and *sir2Δ* strains cultured in YPD medium for up to 72 hours. Cells were collected at the indicated time points and analyzed using anti-HA and anti-Pgk1 antibodies. (C) Subcellular localization of Atg11-GFP in WT and *sir2*Δ strains co-expressing Vph1-mCherry. Cells were cultured in YPD medium for 24 and 72 hours. Scale bar: 5 μm. Data in (A) represent the average of at least three independent experiments (±S.D.).

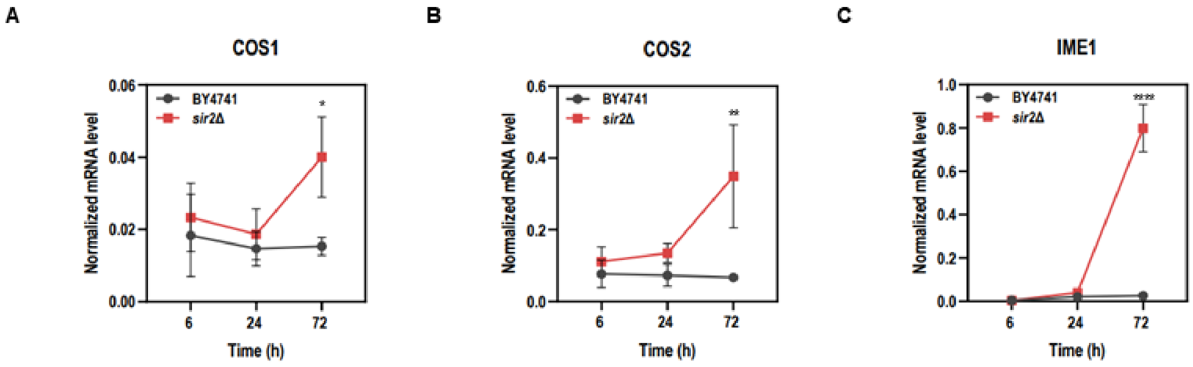

Supplementary Figure 5. (A-C) Quantitative RT-PCR analysis of *COS1* (A)*, COS2* (B)*,* and *IME1* (C) mRNA levels in WT and *sir2*Δ strains cultured in YPD medium for 6, 24, and 72 hours. Expression levels were normalized to *ACT1* mRNA levels. For statistical analysis, the *p*-values were calculated using two-way ANOVA (*$p < 0.05$, **$p < 0.01$, ****$p < 0.0001$). Data represent the average of at least three independent experiments (±S.D.).

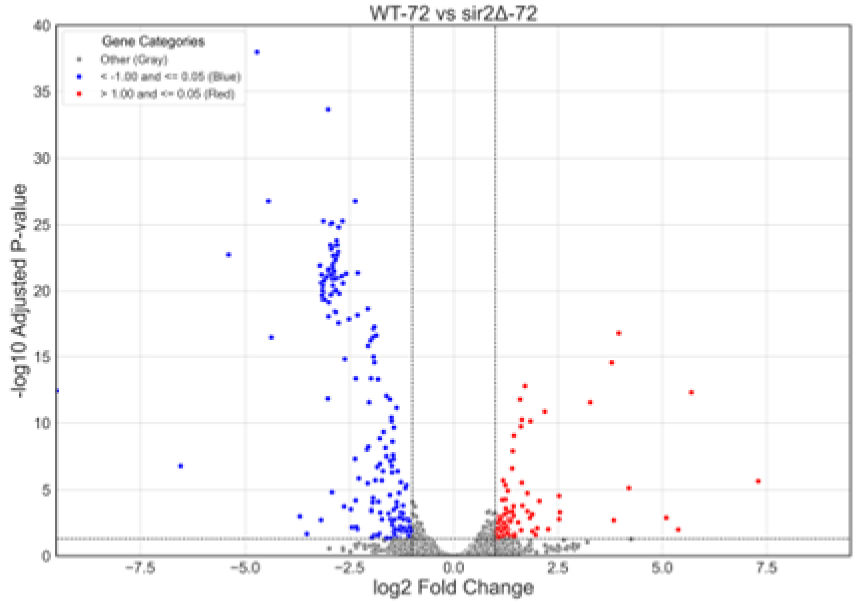

Supplementary Figure 6. Volcano plot of differentially expressed genes in *sir2*Δ compared to WT at 72 hours. Differential expression was determined using the Wald test in DESeq2 with three independent biological replicates per condition. A total of 6,426 genes were analyzed, and 265 genes were identified as significantly differentially expressed (>2-fold change, adjusted p-value < 0.05) in *sir2*Δ cells relative to WT. Red points indicate upregulated genes, blue points indicate downregulated genes, and gray points indicate non-significant genes. The horizontal dashed line marks adjusted $p = 0.05$, and the two vertical dashed lines mark log2 fold change = −1 and +1.

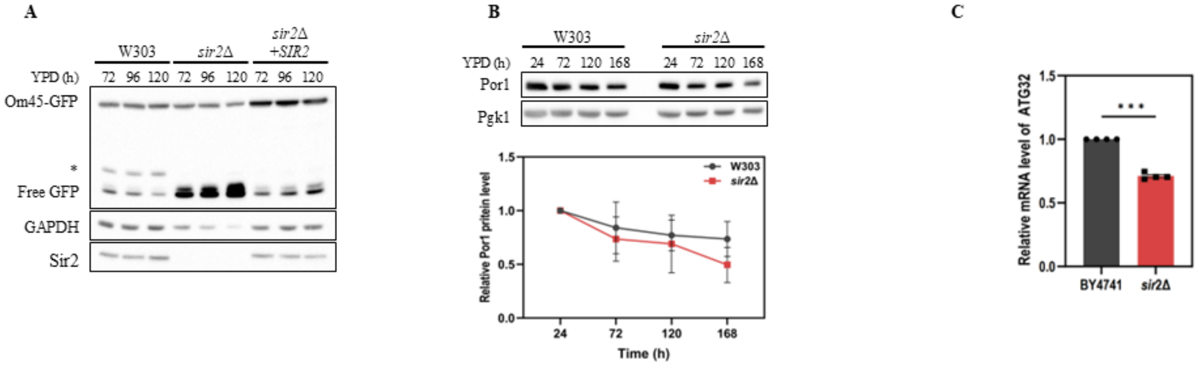

Supplementary Figure 7. Sir2 regulates mitophagy and mitochondrial protein level during the stationary phase. (A) Om45-GFP processing in W303, *sir2*Δ, and *sir2*Δ + *SIR2* strains cultured in YPD medium for up to 72 hours. Cells were collected at the indicated time points and analyzed by immunoblotting using anti-GFP, anti-Sir2, and anti-GAPDH antibodies to assess mitophagy. (B) Relative levels of the mitochondrial outer membrane protein Por1 in WT (W303) and *sir2*Δ strains cultured in YPD medium for up to 168 h. Protein levels were assessed by immunoblotting using anti-Por1 antibodies, with Pgk1 as a loading control. Quantification of three biological replicates showed that Por1 levels declined over time in both strains, with *sir2*Δ exhibiting consistently lower levels than WT ($p < 0.01$ for genotype effect; $p = 0.10$ for genotype × time interaction). (C) Quantitative RT-PCR analysis of *ATG11* mRNA levels in WT and *sir2*Δ strains cultured in YPD medium for 72 hours. For statistical analysis, the *p*-values were calculated using a two-tailed Student's t-test (***$p < 0.001$). Data represent the average of at least three independent experiments (±S.D.).

Strain	Genotype	Reference
BY4741 (Parent strain)	*MATa his3Δ1 leu2Δ0 met15Δ0 ura3Δ0*	Kang *et al*. 2020
sir2Δ	*sir2Δ::tc*	Kang *et al*. 2020
BY4741-rev [GFP-ATG8]	*atg8::GFP-atg8-URA3 his3Δ1::HIS3 YORW22::LEU2 met15Δ::MET15*	in this study
sir2Δ-rev [GFP-ATG8]	*sir2Δ::tc atg8::GFP-ATG8-URA3 his3Δ1::HIS3 YORW22::LEU2 met15Δ::MET15*	in this study
atg1Δ	*atg1Δ::tc*	in this study
sir2Δatg1Δ	*sir2Δ::tc atg1Δ::tc*	in this study
atg11Δ	*atg11Δ::tc*	in this study
atg11Δsir2Δ	*sir2Δ::tc atg11Δ::tc*	in this study
ume6Δ [GFP-ATG8]	*ume6Δ::tc atg8::GFP-atg8-URA3*	in this study
sir2Δume6Δ [GFP-ATG8]	*sir2Δ::tc ume6Δ::tc atg8::GFP-atg8-URA3*	in this study
BY4741 [GFP-ATG8]	*atg8::GFP-atg8-URA3*	in this study
sir2Δ [GFP-ATG8]	*sir2Δ::tc atg8::GFP-ATG8-URA3*	in this study
sir2Δ [SIR2(S473A)-Flag GFP-ATG8]	*sir2Δ::SIR2(S473A)-Flag-tc atg8::GFP-ATG8-URA3*	in this study
sir2Δ [SIR2(S473E)-Flag GFP-ATG8]	*sir2Δ::SIR2(S473E)-Flag-tc atg8::GFP-ATG8-URA3*	in this study
BY4741 [PGK1-GFP]	*pgk1::PGK1-GFP-URA3*	in this study
sir2Δ [PGK1-GFP]	*sir2Δ::tc Pgk1::PGK1-GFP-URA3*	in this study
BY4741 [PGI1-GFP]	*pgi1::PGI1-GFP-URA3*	in this study
sir2Δ [PGI1-GFP]	*sir2Δ::tc Pgi1::PGI1-GFP-URA3*	in this study
BY4741 [OM45-GFP]	*om45::OM45-GFP-URA3*	in this study
sir2Δ [OM45-GFP]	*sir2Δ::tc om45::OM45-GFP-URA3*	in this study
BY4741 [PEX14-GFP]	*pex14::PEX14-GFP-URA3*	in this study
sir2Δ [PEX14-GFP]	*sir2Δ::tc pex14::PEX14-GFP-URA3*	in this study
BY4741 [AMS1-GFP]	*ams1::AMS1-GFP-URA3*	in this study
sir2Δ [AMS1-GFP]	*sir2Δ::tc ams1::AMS1-GFP-URA3*	in this study
BY4741 [RPL25-GFP]	*rpl25::RPL25-GFP-URA3*	in this study
sir2Δ [RPL25-GFP]	*sir2Δ::tc rpl25::RPL25-GFP-URA3*	in this study
BY4741 [FAA4-GFP]	*faa4:FAA4-GFP-URA3*	in this study
sir2Δ [FAA4-GFP]	*sir2Δ::tc faa4::FAA4-GFP-URA3*	in this study
BY4741 [3HA-ATG11]	*atg11::3HA-ATG11-URA3*	in this study
sir2Δ [3HA-ATG11]	*sir2Δ::tc atg11::3HA-ATG11-URA3*	in this study
BY4741 [ATG11-GFP VPH1-mcherry	*atg11::ATG11-GFP-URA3 vph1::VPH1-mcherry-HIS3*	in this study
sir2Δ [ATG11-GFP VPH1-mcherry]	*sir2Δ::tc atg11::ATG11-GFP-URA3 vph1::VPH1-mcherry-HIS3*	in this study
atg11Δ [PEX14-GFP]	*atg11Δ::tc pex14::PEX14-GFP-URA3*	in this study
atg11Δsir2Δ [PEX14-GFP]	*sir2Δ::tc atg11Δ::tc pex14::PEX14-GFP-URA3*	in this study
atg11Δ [AMS1-GFP]	*atg11Δ::tc ams1::AMS1-GFP-URA3*	in this study
atg11Δsir2Δ [AMS1-GFP]	*sir2Δ::tc atg11Δ::tc ams1::AMS1-GFP-URA3*	in this study
atg11Δ [RPL25-GFP]	*atg11Δ::tc rpl25:RPL25-GFP-URA3*	in this study
atg11Δsir2Δ [RPL25-GFP]	*sir2Δ::tc atg11Δ::tc rpl25:RPL25-GFP-URA3*	in this study

atg11Δ [FAA4-GFP]	*atg11Δ::tc faa4:FAA4-GFP-URA3*	in this study
atg11Δsir2Δ [FAA4-GFP]	*sir2Δ::tc atg11Δ::tc faa4:FAA4-GFP-URA3*	in this study
BY4741 [UME6-3HA]	*ume6::UME6-3HA-URA3*	in this study
sir2Δ [UME6-3HA]	*sir2Δ::tc ume6::UME6-3HA-URA3*	in this study

**Strain**	**Genotype**	**Reference**
W303 (Parent strain)	*MAT a/MATα {leu2-3,112 trp1-1 can1-100 ura3-1 ade2-1 his3-11,15} [phi+]*	Kaeberlein lab.
sir2Δ	*sir2Δ::tc*	in this study
sir2Δ [pSIR2-SIR2-Flag]	*sir2Δ::SIR2-Flag-tc*	in this study
atg32Δ	*atg32Δ::tc*	in this study
sir2Δatg32Δ	*sir2Δ::tc atg32Δ::tc*	in this study
W303 [IDH1-GFP]	*idh1::IDH1-GFP-URA3*	in this study
sir2Δ [IDH1-GFP]	*sir2Δ::tc idh1::IDH1-GFP-URA3*	in this study
sir2Δ [SIR2-Flag, IDH1-GFP]	*sir2Δ::SIR2-Flag-tc idh1::IDH1-GFP-URA3*	in this study
sir2Δatg32Δ [IDH1-GFP]	*sir2Δ::tc atg32Δ::tc idh1::IDH1-GFP-URA3*	in this study
sir2Δ [SIR2(H364Y)-Flag, IDH1-GFP]	*sir2Δ::SIR2(H364Y)-Flag-tc idh1::IDH1-GFP-URA3*	in this study
W303 [OM45-GFP]	*om45::OM45-GFP-URA3*	in this study
sir2Δ [OM45-GFP]	*sir2Δ::tc om45::OM45-GFP-URA3*	in this study
sir2Δ [pSIR2-SIR2-Flag OM45-GFP]	*sir2Δ::SIR2-Flag-tc om45::OM45-GFP-URA3*	in this study
W303 [3HA-ATG32]	*atg32::3HA-ATG32-URA3*	in this study
sir2Δ [3HA-ATG32]	*sir2Δ::tc atg32::3HA-ATG32-URA3*	in this study
W303 [VPH1-GFP MTS-mCherry]	*vph1::VPH1-GFP-URA3 his32-11::pTEF1-MTS-mCherry-HIS3*	in this study
sir2Δ [VPH1-GFP MTS-mcherry]	*sir2Δ:: tc vph1::VPH1-GFP-URA3 his32-11::pTEF1-MTS-mCherry-HIS3*	in this study

gene	baseMean	log2FoldCh	lfcSE	stat	pvalue	padj	#NAME?
YFR057W	14.73468	7.299181	1.33459	5.46923	4.52E-08	2.24E-06	5.648802
COS12	54.83933	5.697122	0.731141	7.792097	6.59E-15	4.80E-13	12.31914
YEL073C	7.793453	5.381767	1.524101	3.531109	0.000414	0.010386	1.983548
YCR108C	12.13991	5.095484	1.238793	4.113266	3.90E-05	0.00132	2.879503
YOL166W-	19.32249	4.203772	0.802129	5.24077	1.60E-07	7.57E-06	5.121081
IME1	142.5112	3.960601	0.439025	9.021363	1.86E-19	1.64E-17	16.78586
BSC1	14.84998	3.841849	0.961394	3.996121	6.44E-05	0.002045	2.68922
IMD2	85.07261	3.794107	0.450032	8.430758	3.43E-17	2.69E-15	14.57081
YIR042C	113.294	3.27651	0.433333	7.56118	3.99E-14	2.74E-12	11.56281
PCL1	44.0247	2.548497	0.58542	4.353279	1.34E-05	0.000512	3.290927
PAU9	23.99004	2.529742	0.623706	4.055986	4.99E-05	0.001671	2.777104
YNR077C	42.05509	2.528097	0.50816	4.975001	6.52E-07	2.94E-05	4.532016
MATALPHA	32.35555	2.268038	0.638205	3.553778	0.00038	0.0097	2.013245
VBA3	109.5725	2.185862	0.297665	7.343353	2.08E-13	1.39E-11	10.85799
YHP1	155.5606	2.057189	0.429335	4.791572	1.65E-06	7.19E-05	4.143182
MNC1	23.46811	2.005102	0.555384	3.610298	0.000306	0.008215	2.085418
CLB6	21.65253	1.96719	0.607688	3.237169	0.001207	0.025437	1.594535
GIT1	51.30718	1.887856	0.548275	3.443267	0.000575	0.01378	1.86074
COS5	52.80271	1.884336	0.441855	4.264606	2.00E-05	0.00072	3.142529
SPL2	366.5539	1.842257	0.259075	7.110891	1.15E-12	7.36E-11	10.13298
COS7	58.10302	1.830336	0.444987	4.113236	3.90E-05	0.00132	2.879503
ARO7	47.03573	1.769299	0.402624	4.39442	1.11E-05	0.000432	3.364798
SUC2	124.4517	1.769069	0.349026	5.06858	4.01E-07	1.83E-05	4.737364
DSE4	481.5497	1.712092	0.215841	7.932192	2.15E-15	1.60E-13	12.79478
HXT4	483.1363	1.648753	0.356965	4.618806	3.86E-06	0.000161	3.793119
HMRA2	871.7257	1.637093	0.229037	7.147717	8.82E-13	5.75E-11	10.2402
NCW1	145.3536	1.635503	0.301799	5.419172	5.99E-08	2.93E-06	5.533525
EXG1	3584.411	1.620039	0.462948	3.499397	0.000466	0.011444	1.941428
COS1	511.2903	1.615802	0.231376	6.983457	2.88E-12	1.82E-10	9.739803
HMLALPHA	977.1719	1.593999	0.208978	7.627589	2.39E-14	1.66E-12	11.77907
AAD15	47.60065	1.546283	0.396817	3.896715	9.75E-05	0.002962	2.528399
COS10	67.88426	1.461069	0.446111	3.275126	0.001056	0.022791	1.642237
MSF1	111.9585	1.457838	0.463339	3.146374	0.001653	0.032266	1.491257
YHB1	179.3482	1.452403	0.479494	3.02903	0.002453	0.04253	1.371303
COS3	848.0333	1.445086	0.214091	6.749858	1.48E-11	9.08E-10	9.041933
YLL066W-E	145.0309	1.438152	0.341787	4.207739	2.58E-05	0.000917	3.03756
LSB1	142.7883	1.436119	0.312441	4.596455	4.30E-06	0.000178	3.749335
COS2	872.4622	1.417517	0.223493	6.342559	2.26E-10	1.31E-08	7.88292
SPO24	1975.266	1.406859	0.320677	4.387158	1.15E-05	0.000444	3.352981
MATALPHA	983.6293	1.403508	0.239542	5.85912	4.65E-09	2.47E-07	6.60769
YOL164W-	150.9753	1.372621	0.341426	4.020259	5.81E-05	0.001875	2.726935
PHR1	64.08087	1.368864	0.347343	3.940954	8.12E-05	0.002514	2.599584

PMA1	461.9882	1.345258	0.420138	3.201942	0.001365	0.027826	1.555555
HSP150	790.7546	1.328791	0.335416	3.961623	7.44E-05	0.002341	2.630599
MRPL32	118.5905	1.313332	0.324332	4.049338	5.14E-05	0.001701	2.769378
SRM1	432.8714	1.309649	0.300082	4.3643	1.28E-05	0.00049	3.310138
COS8	592.684	1.301912	0.368863	3.529531	0.000416	0.010386	1.983548
HXT3	285.8792	1.29779	0.251796	5.15413	2.55E-07	1.19E-05	4.92445
GFA1	307.5987	1.286197	0.386881	3.324528	0.000886	0.019937	1.700331
YMR103C	2257.642	1.270126	0.342777	3.705395	0.000211	0.005977	2.223548
NUC1	131.8329	1.257001	0.352192	3.569079	0.000358	0.009302	2.031409
LYS1	261.4536	1.250812	0.277402	4.509026	6.51E-06	0.000263	3.580143
PHO84	677.9082	1.24492	0.256558	4.852393	1.22E-06	5.41E-05	4.266492
COS4	298.248	1.239602	0.232325	5.335627	9.52E-08	4.55E-06	5.34213
DOG2	229.8875	1.236123	0.378278	3.267764	0.001084	0.023232	1.633915
CAR1	676.3186	1.231923	0.28791	4.27885	1.88E-05	0.00068	3.167772
NOP58	517.4092	1.222479	0.339581	3.599965	0.000318	0.008439	2.073695
PAN5	107.7815	1.210023	0.312653	3.870183	0.000109	0.003241	2.489339
CDC3	178.6848	1.193841	0.379371	3.146894	0.00165	0.032266	1.491257
PIN2	371.4652	1.18804	0.216336	5.491647	3.98E-08	1.99E-06	5.700371
RGI1	2407.068	1.168462	0.278755	4.191715	2.77E-05	0.000979	3.009288
YPR015C	533.2987	1.164408	0.240779	4.836012	1.32E-06	5.84E-05	4.233735
ADK2	53.92856	1.161465	0.376039	3.08868	0.00201	0.037176	1.429737
CDC10	129.6785	1.160135	0.296927	3.907143	9.34E-05	0.002851	2.545
RAD59	132.5979	1.151264	0.321399	3.58204	0.000341	0.00889	2.051122
GRS1	1152.024	1.149835	0.308969	3.721529	0.000198	0.005684	2.245312
FKS1	2424.326	1.138049	0.331861	3.429296	0.000605	0.014345	1.843306
TPM2	507.9576	1.130527	0.274709	4.115368	3.87E-05	0.00132	2.879503
PNP1	247.6248	1.106354	0.275679	4.013199	5.99E-05	0.001922	2.716163
COS6	403.4288	1.100696	0.272516	4.039021	5.37E-05	0.001749	2.757095
CIR1	195.6809	1.088715	0.261624	4.161365	3.16E-05	0.0011	2.958668
ATG41	1206.293	1.085257	0.350969	3.092173	0.001987	0.036898	1.433003
SWI6	384.7027	1.072961	0.276726	3.877347	0.000106	0.003162	2.50004
SET5	70.0439	1.069155	0.339941	3.145116	0.00166	0.032266	1.491257
POL1	1012.258	1.067932	0.297165	3.593737	0.000326	0.008571	2.066966
YPK2	274.2074	1.06193	0.352756	3.010377	0.002609	0.044511	1.351536
MCM2	340.216	1.042244	0.322201	3.234766	0.001217	0.025566	1.592339
ABP1	1603.748	1.037297	0.308532	3.362037	0.000774	0.017866	1.747963
DCP1	77.13755	1.029491	0.335789	3.065891	0.00217	0.038915	1.409885
SLK19	2101.734	1.028987	0.333202	3.088182	0.002014	0.037176	1.429737
TUB2	237.5826	1.018387	0.300152	3.392901	0.000692	0.016029	1.795094
DYS1	381.5073	1.013333	0.287286	3.527256	0.00042	0.010386	1.983548
YNR065C	501.2598	1.012553	0.30501	3.319736	0.000901	0.020138	1.695984
BFR1	983.8477	1.012093	0.314352	3.219618	0.001284	0.026676	1.573877
COG7	872.9105	1.004766	0.253253	3.967439	7.26E-05	0.002296	2.638999

ENB1	110.7988	-1.01687	0.293687	-3.46243	0.000535	0.012885	1.889918
CUE2	78.63744	-1.031	0.334301	-3.08403	0.002042	0.037372	1.427453
DAS2	95.32935	-1.0311	0.335583	-3.07256	0.002122	0.038497	1.414577
RME3	129.8292	-1.04687	0.322462	-3.2465	0.001168	0.024701	1.607288
SNZ3	126.3466	-1.05781	0.339043	-3.11999	0.001809	0.034451	1.462799
YNL284C-/	267.7266	-1.06116	0.288533	-3.67777	0.000235	0.006544	2.18416
RPS6B	1135.562	-1.0704	0.341861	-3.13109	0.001742	0.033535	1.474495
YPQ1	218.4311	-1.07393	0.271201	-3.95989	7.50E-05	0.002346	2.629625
MTG1	3731.033	-1.08637	0.349313	-3.11	0.001871	0.035053	1.455273
ZIP2	141.2756	-1.09562	0.336525	-3.25568	0.001131	0.024161	1.616887
ATS1	444.9431	-1.10189	0.271293	-4.06161	4.87E-05	0.00164	2.785235
FRE3	244.6922	-1.12385	0.32389	-3.46986	0.000521	0.012631	1.898569
RME2	1072.059	-1.13365	0.212433	-5.33648	9.48E-08	4.55E-06	5.34213
XBP1	1330.67	-1.15554	0.220534	-5.23972	1.61E-07	7.57E-06	5.121081
YEL009C-A	133.3146	-1.18545	0.325557	-3.64129	0.000271	0.007349	2.133762
HSP12	11074.68	-1.20334	0.297144	-4.04968	5.13E-05	0.001701	2.769378
MRK1	2621.536	-1.21304	0.262239	-4.62571	3.73E-06	0.000157	3.804671
FUS2	152.2312	-1.21684	0.282099	-4.31354	1.61E-05	0.000595	3.225538
HES1	221.3919	-1.21928	0.338362	-3.60347	0.000314	0.008397	2.075855
TKL2	369.6222	-1.23117	0.345843	-3.55989	0.000371	0.009517	2.021512
TDH1	8279.154	-1.23402	0.358539	-3.44179	0.000578	0.013803	1.860034
ECM10	327.1914	-1.23598	0.298407	-4.14192	3.44E-05	0.001191	2.924174
PDR3	261.758	-1.26657	0.23305	-5.43475	5.49E-08	2.70E-06	5.568
SRB2	341.6548	-1.27072	0.292426	-4.34543	1.39E-05	0.000524	3.280651
THI7	102.6362	-1.27483	0.367611	-3.46788	0.000525	0.012675	1.897044
DIA3	238.998	-1.27917	0.344968	-3.70807	0.000209	0.005941	2.226157
AHC2	297.9665	-1.29001	0.427254	-3.0193	0.002534	0.043678	1.359736
RPL26B	968.9268	-1.29961	0.31513	-4.12404	3.72E-05	0.00128	2.892763
YOR343W-	67.42827	-1.3082	0.370734	-3.52866	0.000418	0.010386	1.983548
RPL27A	606.3472	-1.30941	0.257941	-5.07637	3.85E-07	1.77E-05	4.751976
STE5	363.9399	-1.34378	0.232731	-5.77395	7.74E-09	4.04E-07	6.393824
RBH2	112.5035	-1.36724	0.287783	-4.75092	2.02E-06	8.68E-05	4.061516
SGA1	388.1485	-1.36759	0.298846	-4.57624	4.73E-06	0.000192	3.715841
ESF1	2442.338	-1.36828	0.184025	-7.43531	1.04E-13	7.02E-12	11.15367
SOR1	31.0773	-1.38783	0.454675	-3.05236	0.002271	0.040013	1.397799
STE4	160.005	-1.40427	0.397985	-3.52843	0.000418	0.010386	1.983548
TIR4	244.8901	-1.41943	0.431893	-3.28654	0.001014	0.022039	1.656803
YOR186W	170.8511	-1.42523	0.353426	-4.03261	5.52E-05	0.001789	2.747498
SHC1	126.5957	-1.42725	0.396421	-3.60035	0.000318	0.008439	2.073695
HXT10	87.31334	-1.43097	0.390985	-3.65992	0.000252	0.006911	2.160456
ASP3-4	1983.66	-1.43412	0.206188	-6.95541	3.52E-12	2.20E-10	9.657598
HO	93.40084	-1.43734	0.321975	-4.46414	8.04E-06	0.00032	3.49435
SMA2	366.5561	-1.44103	0.420754	-3.42488	0.000615	0.014525	1.837888

STE18	252.2158	-1.44212	0.235289	-6.12915	8.83E-10	4.94E-08	7.30658
ECM33	5570.401	-1.4531	0.232977	-6.23711	4.46E-10	2.56E-08	7.591922
OPI3	2943.47	-1.46776	0.222471	-6.59755	4.18E-11	2.52E-09	8.599423
GAP1	72.68181	-1.47106	0.373511	-3.93847	8.20E-05	0.002528	2.597233
YHR210C	51.49796	-1.4754	0.389821	-3.78481	0.000154	0.004457	2.350986
KNH1	474.8097	-1.47686	0.257304	-5.73977	9.48E-09	4.90E-07	6.30951
MEP2	237.9928	-1.48335	0.249954	-5.9345	2.95E-09	1.60E-07	6.794821
ASP3-3	1930.16	-1.48498	0.208751	-7.11363	1.13E-12	7.29E-11	10.13713
ASP3-1	1916.134	-1.49058	0.206937	-7.20308	5.89E-13	3.88E-11	10.4114
YMR262W	44.36329	-1.50119	0.501645	-2.99253	0.002767	0.046544	1.332141
RPS27A	1034.972	-1.52203	0.250353	-6.07953	1.21E-09	6.68E-08	7.175529
THI4	262.6466	-1.52221	0.376364	-4.04452	5.24E-05	0.001727	2.762824
ASP3-2	1917.097	-1.52791	0.200181	-7.63264	2.30E-14	1.62E-12	11.79125
GAL1	161.7028	-1.52862	0.305361	-5.00597	5.56E-07	2.52E-05	4.598503
THI22	423.2113	-1.56157	0.363502	-4.29591	1.74E-05	0.00064	3.193511
COA3	57.47862	-1.60661	0.534627	-3.0051	0.002655	0.045026	1.34654
MEP3	1037.654	-1.61104	0.208965	-7.70961	1.26E-14	9.08E-13	12.04202
GAL80	344.6007	-1.61217	0.259869	-6.20378	5.51E-10	3.14E-08	7.503632
RPL17B	2954.734	-1.63406	0.254043	-6.43219	1.26E-10	7.43E-09	8.129257
CTT1	1227.86	-1.68025	0.245473	-6.84495	7.65E-12	4.74E-10	9.324199
RPS27B	592.6008	-1.69438	0.293164	-5.77962	7.49E-09	3.94E-07	6.404812
LEE1	375.8339	-1.71948	0.425261	-4.04334	5.27E-05	0.001727	2.762824
YGR201C	1796.692	-1.7227	0.313615	-5.49304	3.95E-08	1.99E-06	5.700371
YGL188C-/	105.7012	-1.75985	0.406141	-4.33311	1.47E-05	0.000551	3.258917
YMR046C	157.5289	-1.76765	0.294965	-5.99275	2.06E-09	1.13E-07	6.945934
RPL33B	830.9442	-1.77077	0.265057	-6.68074	2.38E-11	1.44E-09	8.840299
YNL146W	24.67183	-1.78258	0.538128	-3.31256	0.000924	0.020588	1.686382
XYL2	50.21888	-1.80525	0.37872	-4.76673	1.87E-06	8.08E-05	4.092526
YDR034C-[	584.8467	-1.8143	0.224613	-8.07744	6.61E-16	4.99E-14	13.30218
YOL155W-	19.37671	-1.81528	0.566145	-3.20638	0.001344	0.027579	1.559417
RME1	91.52332	-1.83305	0.310592	-5.90181	3.60E-09	1.92E-07	6.716016
YDR261W-	641.41	-1.85644	0.206864	-8.97424	2.85E-19	2.48E-17	16.60557
YGR109W-	72.76084	-1.87804	0.341484	-5.49965	3.81E-08	1.94E-06	5.713073
YOR343W-	639.9479	-1.90169	0.225395	-8.43713	3.25E-17	2.58E-15	14.58903
YOR192C-I	669.2149	-1.90242	0.20806	-9.14363	6.04E-20	5.48E-18	17.26146
YFR018C	41.95094	-1.91428	0.599716	-3.19198	0.001413	0.028617	1.543382
YFL002W-/	699.2957	-1.92102	0.224661	-8.55075	1.22E-17	9.94E-16	15.00265
YBL100W-I	675.2012	-1.92526	0.211407	-9.10692	8.48E-20	7.58E-18	17.12048
UTH1	1076.946	-1.93291	0.394065	-4.90506	9.34E-07	4.17E-05	4.379356
YDR210W-	669.1974	-1.93558	0.216443	-8.94269	3.80E-19	3.26E-17	16.4873
RPL25	243.5253	-1.94361	0.410452	-4.7353	2.19E-06	9.25E-05	4.033906
RPS19A	67.05447	-1.95753	0.643526	-3.04187	0.002351	0.041098	1.386174
snR39	37.26986	-1.97046	0.446776	-4.41041	1.03E-05	0.000406	3.391378

MHF2	43.15243	-1.97084	0.441612	-4.46283	8.09E-06	0.00032	3.49435
YLR410W-I	608.4932	-1.98111	0.244563	-8.10062	5.47E-16	4.22E-14	13.37421
YGR109W-	391.6838	-1.99148	0.224404	-8.87455	7.02E-19	5.86E-17	16.23217
YBL005W-/	178.916	-2.0284	0.268297	-7.56025	4.02E-14	2.74E-12	11.56281
PDC6	192.2757	-2.04713	0.316676	-6.46444	1.02E-10	6.06E-09	8.217534
YGR161W-	691.4298	-2.05311	0.234176	-8.76735	1.83E-18	1.51E-16	15.82211
YCL019W	659.3683	-2.05883	0.216972	-9.48893	2.33E-21	2.36E-19	18.62784
YLR030W	283.7479	-2.06096	0.381679	-5.39972	6.67E-08	3.24E-06	5.489742
YER138W-/	158.7869	-2.07608	0.324967	-6.38858	1.67E-10	9.79E-09	8.009114
NEJ1	82.16957	-2.27448	0.409138	-5.5592	2.71E-08	1.39E-06	5.856928
YIL082W-A	523.8297	-2.29981	0.225771	-10.1865	2.28E-24	4.60E-22	21.3372
SDD1	678.827	-2.30617	0.246191	-9.36739	7.43E-21	7.16E-19	18.1452
OSW1	22.31621	-2.30831	0.647385	-3.56559	0.000363	0.009388	2.027432
MF(ALPHA	18.19257	-2.33319	0.634856	-3.67515	0.000238	0.006582	2.181625
FAR1	286.625	-2.34682	0.289836	-8.09704	5.63E-16	4.30E-14	13.36678
AGA2	106.94	-2.3488	0.487813	-4.81497	1.47E-06	6.44E-05	4.190926
AXL1	728.9085	-2.35919	0.204911	-11.5133	1.13E-30	1.74E-27	26.75904
ASG7	62.53166	-2.36312	0.384943	-6.13888	8.31E-10	4.69E-08	7.329264
DGR1	18.32234	-2.43802	0.666251	-3.65931	0.000253	0.006911	2.160456
YLR031W	197.4567	-2.46497	0.550358	-4.47885	7.50E-06	0.000301	3.521351
YNL054W-	1388.821	-2.51023	0.270203	-9.29019	1.54E-20	1.44E-18	17.84207
YHR214C-(	1788.038	-2.57848	0.253551	-10.1695	2.71E-24	5.31E-22	21.27516
STE2	137.2187	-2.60978	0.30688	-8.50424	1.83E-17	1.47E-15	14.8337
GAL10	21.79451	-2.62745	0.572604	-4.58859	4.46E-06	0.000184	3.735838
YBR012W-.	1580.095	-2.65265	0.265992	-9.97267	2.01E-23	2.79E-21	20.5541
YML045W-	1779.635	-2.66493	0.238451	-11.176	5.34E-29	5.57E-26	25.25385
YER159C-A	1727.76	-2.6827	0.265327	-10.1109	4.94E-24	8.14E-22	21.0894
YPR158W-.	1655.281	-2.74265	0.280664	-9.77203	1.48E-22	1.69E-20	19.77122
YOR142W-	1661.199	-2.75699	0.249897	-11.0325	2.66E-28	1.67E-25	24.77819
YMR045C	7598.688	-2.76113	0.299508	-9.21888	3.00E-20	2.76E-18	17.55864
YDR098C-/	1731.916	-2.77394	0.261862	-10.5932	3.21E-26	1.18E-23	22.92812
YMR051C	1751.249	-2.78095	0.259316	-10.7242	7.84E-27	3.65E-24	23.43805
YOL103W-	2325.779	-2.79358	0.260483	-10.7246	7.80E-27	3.65E-24	23.43805
YDR316W-	1754.117	-2.79512	0.265567	-10.5251	6.62E-26	2.18E-23	22.66161
YAR010C	1879.116	-2.79796	0.277834	-10.0706	7.45E-24	1.17E-21	20.93333
YDR098C-E	6908.265	-2.80743	0.259523	-10.8177	2.84E-27	1.62E-24	23.79174
YGR161C-(	1729.088	-2.82261	0.286852	-9.83995	7.57E-23	9.29E-21	20.03178
YPR137C-A	1836.886	-2.82627	0.299881	-9.42463	4.32E-21	4.22E-19	18.37466
YNL284C-E	7062.573	-2.82992	0.270974	-10.4435	1.57E-25	4.68E-23	22.33006
YML040W	2274.996	-2.84993	0.301944	-9.43862	3.78E-21	3.75E-19	18.42573
YGR038C-/	1711.977	-2.8585	0.284189	-10.0584	8.43E-24	1.29E-21	20.89042
YGR161C-[	7646.775	-2.8603	0.291141	-9.82446	8.83E-23	1.06E-20	19.97338
YPR158C-(	2420.506	-2.86752	0.280721	-10.2148	1.70E-24	3.63E-22	21.43994

YBR012W-	9756.587	-2.87327	0.284047	-10.1155	4.72E-24	7.98E-22	21.09813
YBL005W-	8438.585	-2.88303	0.277639	-10.3841	2.93E-25	8.33E-23	22.07911
YDR210C-	7111.977	-2.88958	0.274777	-10.5161	7.28E-26	2.28E-23	22.64235
YOR142W-	10198.77	-2.89589	0.281644	-10.2821	8.49E-25	2.04E-22	21.68974
YPL257W-	2306.183	-2.89712	0.291646	-9.93368	2.97E-23	4.01E-21	20.39721
YDR210C-	1472.039	-2.90192	0.271351	-10.6944	1.08E-26	4.51E-24	23.34561
YER137C-A	2368.711	-2.90518	0.280965	-10.34	4.64E-25	1.22E-22	21.9124
YER160C	8882.941	-2.91112	0.289632	-10.0511	9.09E-24	1.35E-21	20.86841
YDR261C-	1881.93	-2.9165	0.298462	-9.77176	1.49E-22	1.69E-20	19.77122
YML045W	9169.882	-2.91706	0.262374	-11.118	1.03E-28	8.03E-26	25.09553
YLR157W-	32.43771	-2.91761	0.571702	-5.10337	3.34E-07	1.55E-05	4.810605
YHR214C-	10080.71	-2.92342	0.28764	-10.1635	2.89E-24	5.47E-22	21.2618
YPR158W-	8940.553	-2.93308	0.27554	-10.6448	1.84E-26	7.21E-24	23.14219
YLR157C-A	2374.358	-2.93401	0.289291	-10.1421	3.59E-24	6.43E-22	21.19206
YJR028W	1903.695	-2.94504	0.30205	-9.75017	1.84E-22	2.06E-20	19.68658
YGR027W-	2298.029	-2.95015	0.288873	-10.2126	1.74E-24	3.63E-22	21.43994
YDR316W-	9052.967	-2.9538	0.275529	-10.7205	8.16E-27	3.65E-24	23.43805
YGR038C-	8584.505	-2.954	0.266289	-11.0932	1.35E-28	9.41E-26	25.02635
YDR365W-	2299.15	-2.96836	0.293077	-10.1283	4.14E-24	7.20E-22	21.14296
YLR227W-	2292.003	-2.99481	0.311711	-9.60765	7.42E-22	7.61E-20	19.11837
YLR256W-	2267.704	-3.00241	0.321386	-9.34206	9.45E-21	8.96E-19	18.04778
YJR026W	2283.122	-3.00417	0.293905	-10.2216	1.59E-24	3.55E-22	21.44993
YPR158C-	10754	-3.00676	0.293094	-10.2587	1.08E-24	2.51E-22	21.60083
YDR366C	801.2217	-3.01095	0.234647	-12.8318	1.09E-37	2.27E-34	33.64414
SRD1	104.8426	-3.01635	0.394253	-7.65081	2.00E-14	1.42E-12	11.84764
YNL054W-	14905.68	-3.04979	0.302148	-10.0937	5.89E-24	9.45E-22	21.02444
YMR050C	14700.36	-3.09386	0.320356	-9.65759	4.56E-22	4.81E-20	19.3176
YPR137C-	17032.06	-3.10777	0.309998	-10.0251	1.18E-23	1.72E-21	20.76444
YLR035C-A	5134.651	-3.12297	0.279788	-11.1619	6.26E-29	5.60E-26	25.25184
YJR029W	16764.77	-3.12369	0.319006	-9.79195	1.22E-22	1.44E-20	19.84178
YOL103W-	18692.84	-3.13523	0.315656	-9.9324	3.01E-23	4.01E-21	20.39721
YGR027W-	17116.66	-3.14086	0.32526	-9.65648	4.61E-22	4.81E-20	19.3176
YPL257W-	18667.8	-3.14441	0.319485	-9.8421	7.41E-23	9.28E-21	20.03244
YAR009C	14119.71	-3.14813	0.317409	-9.9182	3.47E-23	4.52E-21	20.34453
YLR157C-B	19517.4	-3.15197	0.323757	-9.73559	2.13E-22	2.30E-20	19.63848
YER138C	19474.06	-3.1529	0.320254	-9.84498	7.21E-23	9.20E-21	20.03611
YML039W	18579.65	-3.15467	0.324042	-9.73536	2.13E-22	2.30E-20	19.63848
YDR261C-	19319.95	-3.16391	0.311838	-10.146	3.45E-24	6.35E-22	21.19696
YJR027W	18235.07	-3.17566	0.318106	-9.98302	1.81E-23	2.57E-21	20.58964
YCL021W-	19.00663	-3.18042	0.793679	-4.00719	6.14E-05	0.001962	2.70733
YDR365W-	19460.97	-3.19584	0.309104	-10.339	4.69E-25	1.22E-22	21.9124
YLR227W-	12958.7	-3.20867	0.310688	-10.3276	5.28E-25	1.32E-22	21.87847
MFA1	297.5077	-3.51952	1.069427	-3.29104	0.000998	0.021768	1.662189

tN(GUU)P	13.27639	-3.68643	0.882437	-4.17755	2.95E-05	0.001036	2.984658
BAR1	135.8841	-4.3695	0.489006	-8.93548	4.05E-19	3.43E-17	16.46488
IRT1	2361.675	-4.44209	0.386423	-11.4954	1.39E-30	1.74E-27	26.75904
ICS2	720.3576	-4.71314	0.34621	-13.6135	3.33E-42	1.04E-38	37.98248
YPR027C	90.02631	-5.39571	0.511863	-10.5413	5.57E-26	1.94E-23	22.71283
YGL193C	527.8991	-5.48342	0.310149	-17.6799	5.99E-70	3.75E-66	65.42641
MFA2	660.989	-6.52603	1.100432	-5.93042	3.02E-09	1.63E-07	6.787795
SIR2	125.6001	-9.50799	1.214651	-7.82775	4.97E-15	3.66E-13	12.43692
